# Supplementary material for: Temporal measures of oropharyngeal swallowing events identified using ultrasound imaging in healthy young adults
Source: PLoS One. 2022 Jun 28;17(6):e0270704. doi: 10.1371/journal.pone.0270704 (PMC9239467; doi:10.1371/journal.pone.0270704)
Supplement: Attachment — Submitted filename: Responses to reviewers_20200201.docx [file pone.0270704.s002.docx]

**Attachment – Responses to reviewers’ comments**

**Reviewer #1:**

1) The study is interesting as well as the well-executed statistical analysis. However, to improve the quality of the manuscript I recommend to insert these following references:

   - In the introduction emphasizes the role of Patient-reported measures in dysphagia as illustrated in an interesting systematic review indicated: Patel DA, Sharda R, Hovis KL, Nichols EE, Sathe N, Penson DF, Feurer ID, McPheeters ML, Vaezi MF, Francis DO. Patient-reported outcome measures in dysphagia: a systematic review of instrument development and validation. Dis Esophagus. 2017 May 1;30(5):1-23. doi: 10.1093/dote/dow028.

   - In the introduction to suggest other methods of evaluating swallowing movement using ultrasonography: Matsuo T, Mstsuyama M, Nakatani K, Naoe M. Evaluation of swallowing movement using ultrasonography. Radiological Physics and Technology .2019 May; 13(1):62-68. doi:10.1007/s12194-019-00547-1

   - In the discussion add a systematic review of hyoid and laryngeal movements as indicated in the following reference:
   Sonja M. Molfenter, Catriona M. Steele. Physiological variability in the deglutition literature: hyoid and laryngeal kinematics. Dysphagia. 2011 Mar;26(1):67-74. doi: 10.1007/s00455-010-9309-x.

*Response:*

Thank you for your assuring comments. The alternative methods used by Matsuo and colleagues (2019) is now included in the third paragraph of Introduction, whereas the review by Molfenter and Steele (2011) is now included in the paragraph before “Limitations and recommendations” in Discussion. However, the systematic review on patient-reported outcome measures by Patel and colleagues (2017) may, with all due respect, not be deemed relevant to the present study. The authors found it difficult to be included in the Introduction section.

**Reviewer #2:**

The study invetigated the time sequence of hyoid bone and tongue base movement by ultrasound when swallowing different volume and consistency of bolus. Obligatory and non-obligator sequence was identified and variability of adherence to several sequence was reported.
The topic is of clinical significance. However, several issues should be addressed and clarified to validate the study.

1) The procedure of ultrasound exam should be specified in detail, such as the head support, placement and fixation of gel pad, transducer holding, because the stabilization of transducer and image quality is critical for accurate analysis of hyoid and tongue movemnent.

*Response:*

More details regarding the procedures of ultrasound exam are added to the section “Ultrasound image acquisition”. Readers are referred to a published study for figures showing the experimental set up. Figures are not provided in this manuscript in light of the copyright issues. The paragraph now reads:

*“Subjects sat comfortably in an upright position with their head supported against a wall or with manual support by the examiner throughout the image acquisition process. The ultrasound transducer was placed on the mid-sagittal plane in the submental area of subjects. Readers may refer to figures of a previous study [**23**] for the placement and fixation of gel pads and the transducer”*

2) US is highly operator dependent. Is the operator experienced with US exam? The inadequate reliability of US would make the results doutful.

*Response:*

The background of the operators are now detailed in the section “Ultrasound image acquisition” as follows:

*“All ultrasound images were acquired by two final-year Master of Speech Therapy students who were trained to manage dysphagia (one of them is the second author P.S.). Before the commencement of image acquisition, both examiners were trained by a research personnel, who had at least three years of experience in conducting ultrasonographic data collection,on the operation of the ultrasound system and had gone through at least ten hours of practice on a number of individuals.”*

3) Are the subjects holding the bolus at mouth floor or in front of the tongue? This may significantly alter the results.

*Response:*

In the second paragraph of the section “Ultrasound image acquisition”, how the subjects were fed the bolus and how they hold the bolus in the anterior part of the tongue surface are now specified:

*“In each trial, subjects were fed the designated bolus using a syringe. They were required to hold the bolus in the anterior part of the tongue surface before being instructed to swallow by the examiners.”*

4) The localization of hyoid and tongue base in sonographic view should be elaborated.
   In figure 1, the B indicate the acoustic shadow but not hyoid bone. The presence of acoustic shadow is extremely variable depending on the placement position and angle of transducer, therefore, should not be used for quantitative analysis. The margin of hyoid bone or the interface between hyoid bone/geniohyoid bone should be used as a consistent marker.

*Response:*

Thank you for your comment. The figure is now re-annotated accordingly.

5) A clear definition of event, such as onset and offset of their movement is required.

*Response:*

The definitions below are added to the first paragraph of the section “Data extraction”:

*“Displacement onset” was defined as the point at which the corresponding structure started to leave its at-rest position and commence movement. “Maximum displacement” was defined as the point at which the corresponding structure reached the position farthest away from its at-rest position. “Displacement offset” was defined as the point at which the corresponding structure started to return to its at-rest position from the maximally displaced position.*

6) Several previous studies have investigated the time sequence of swallowing events in relation to bolus consistency and volume, including some ultrasound studies. The introduction should briefly review these studies and point out what is lacking.

*Response:*

On top of the studies by McConnel et al (1998) and Mendell and Logemann (2007) and Molfenter and Steele (2012), two more studies are now reviewed in the second last paragraph of Introduction. They are:

- Nagy A, Molfenter SM, Péladeau-Pigeon M, Stokely S, Steele CM. The Effect of Bolus Volume on Hyoid Kinematics in Healthy Swallowing. Biomed Res Int. 2014;2014:738971-6.
- Nagy A, Molfenter SM, Péladeau-Pigeon M, Stokely S, Steele CM. The Effect of Bolus Consistency on Hyoid Velocity in Healthy Swallowing. Dysphagia. 2015;30(4):445-51.

The gaps in the literature are highlighted in the last paragraph of Introduction as:

*“To the best of the authors’ knowledge, this study is the first to investigate swallowing events, and also their variability, related to tongue base retraction and hyoid bone excursion collectively using ultrasonography. Effects of bolus property on swallowing events captured on ultrasound images had not been previously reported in the literature. Further, this study does not only focus on the onset of the two swallowing movements, but also their maximum displacement and offset as swallowing events.”*

7) As the authors pointed out in the limitations, only one trial of swallow is analysed. This would significantly affect the validity of the study as large between-trial variation exists even in the same individual.

*Response:*

We acknowledge that between-trial variability may exist. However, as this is the very first study to investigate the subject matter, it may be difficult to take into account all sources of variability. Readers are, nevertheless, alerted for such variability in the Limitations and recommendations section.

8) Table 2 & 3: are there any differences betwen groups?

*Response:*

A series of two-way ANOVAs were conducted to look into the effects of bolus volume and consistency on the timing of events. Results are summarized in the newly-numbered Table 3. The paragraph below is added in the Result section to summarize the results of the ANOVAs:

*“Table 3 shows the results of two-way ANOVAs on the time difference between HBOn and the corresponding events. All interaction effects and main effects of bolus volume were statistically non-significant. On the contrary, main effects of bolus consistency for all events were significant (p ≤ 0.033), with the exception of TBOn (F(1, 157) = 2.674, p = 0.104, η_p_^2^ = 0.017). Effect sizes for the main effect of bolus consistency were small for TBMax (η_p_^2^ = 0.029) and TBOff (η_p_^2^ = 0.033) and medium for HBMax (η_p_^2^ = 0.066) and HBOff (η_p_^2^ = 0.065).”*

The following paragraph is also added to Discussion to interpret the abovementioned results:

*“Significant main effects from the ANOVAs suggested that the timing at which HBMax, TBMax, HBOff, and TBOff occur may be influenced by bolus consistencies, with thick boluses resulting in earlier occurrence of these events than thin boluses. A previous study by Nagy et al. stated that thicker boluses would result in faster hyoid bone movements [22]. In the present study, this may be reflected by arriving at the maximum hyoid bone displacement at an earlier time point. Since the timing of swallowing events was measured by the time difference from a reference event (i.e. HBOn, the first-occurring event), the significant difference in the timing of HBMax itself may account for differences in timing for any subsequent events. Despite the main effect of bolus consistency was found in TBMax, HBOff and TBOff, it is inconclusive to claim that the significant differences in timing of these events were resulted from thicker boluses. On the other hand, the non-significant volume effect on the timing of hyoid bone-related events echoes with another study by Nagy and colleagues, which also found that difference in bolus volume did not necessarily lead to difference in hyoid bone displacement velocity [21]. The timing of HBMax and any subsequent relevant events may, therefore, not differ with bolus volume.”*

Regarding the data presented in the original Table 3 (now numbered Table 4), the effects of bolus type on level of adherence were analyzed with Pearson’s Chi Squared tests and the results are summarized and interpreted in the Results and Discussion sections respectively as follows:

*“In all pairwise bolus type comparisons in level of adherence, the only significant difference was found between 5mL x thin and 10mL x thick boluses in the HBMax < TBMax sequence (χ^2^ = 5.020, df = 1, N = 80 , p = 0.025; phi = -0.250).”*

*“It is interesting to note that from the largely non-significant pairwise comparisons, bolus type had little or no effect on the adherence to the anticipated sequences of paired-events.”*

9) Table 4. The effect of bolus volume and consistency should be considered separately.

*Response:*

The analyses summarized in the original Table 4 (now Table 5) were to look into the association between bolus type and adherence, instead of how individual bolus type may affect adherence. With all due respect, it may deem inappropriate to consider the bolus type separately.

10) The discussion should include more comparison with previous studies, instead of repeat of results. For example, how bolus consistenct affect movement sequence of hyoid bone and tongue base.

*Response:*

In the Discussion section, results are now interpreted in relation to the following studies. How bolus volume and consistency may affect temporal aspects of swallowing events is further discussed.

- Mendell DA, Logemann JA. Temporal Sequence of Swallow Events During the Oropharyngeal Swallow. J Speech Lang Hear Res. 2007;50(5):1256-71.
- Molfenter SM, Steele CM. Temporal Variability in the Deglutition Literature. Dysphagia. 2012;27(2):162-77.
- Nagy A, Molfenter SM, Péladeau-Pigeon M, Stokely S, Steele CM. The Effect of Bolus Volume on Hyoid Kinematics in Healthy Swallowing. Biomed Res Int. 2014;2014:738971-6.
- Nagy A, Molfenter SM, Péladeau-Pigeon M, Stokely S, Steele CM. The Effect of Bolus Consistency on Hyoid Velocity in Healthy Swallowing. Dysphagia. 2015;30(4):445-51.
