# Supplementary material for: Temporal measures of oropharyngeal swallowing events identified using ultrasound imaging in healthy young adults
Source: PLoS One. 2022 Jun 28;17(6):e0270704. doi: 10.1371/journal.pone.0270704 (PMC9239467; doi:10.1371/journal.pone.0270704)
Supplement: Attachment — Submitted filename: Response to reviewers_20220519.docx [file pone.0270704.s004.docx]

**Attachment – Responses to reviewer**

Reviewer #3: The present study was designed to establish the temporal property of swallowing events using ultrasound imaging and to investigate the variability of the sequences and effect of bolus type on it in healthy individuals. The study on ultrasound imaging is interesting, but some concerns are raised mainly with respect to the methods.

*Response:*

*Thank you for reviewing the manuscript. Please see the point-to-point responses to your comments as follows.*

1) P3, L8 – What is “four stages”? The authors are recommended to explain this term in brief because not all readers are familiar with swallowing physiology.

*Response:*

*Thank you for your suggestion. We have included a brief description on the four swallowing stages at the beginning of the second paragraph under “Introduction”:*

*“The process of swallowing is commonly divided into four stages; namely, 1) the oral preparatory state which involves mostly mastication, 2) the oral stage which involves propulsion of the processed bolus towards the oropharynx, 3) the pharyngeal stage which starts when the bolus reaches the anterior faucial arch and ends when the bolus has entirely passed the upper esophageal sphincter (UES), and 4) the esophageal stage in which the bolus travels along the esophagus before reaching the lower esophageal sphincter.”*

2) P3, L6 – from the bottom, Patients with radiotherapy
   Do the authors mean “patients with head and neck cancer”?

*Response:*

*Indeed. We have now specified as “*Head and neck cancer patients who had undergone radiotherapy”. The sentence now reads:

*“Head and neck cancer patients who had undergone radiotherapy were observed to have delayed hyoid bone movement relative to pharyngeal bolus transit, yet it was compensated by an earlier arrival of maximum hyoid bone movement [**6**].”*

3) Materials and methods, Procedure
   P6, Materials and equipment
   How was the sampling rate of recordings?

*Response:*

*We hope we have not mistaken your comment. To our understanding, sampling rate is primarily referring to recording of audio signals. For recording of images, it is stated under the section “Ultrasound image acquisition” that:*

*“Ultrasound images were recorded at a fixed frame rate of 32 frames per second…”*

4) P8, L2 – The authors selected “the best image” of the three data. How did the authors determine the best? In addition, were there any variation of time sequence across the three images? This may be helpful to interpret reproducibility and reliability.

*Response:*

*The recorded trials with the best image quality (i.e. with the highest clarity and least artifacts) were selected for imaging processing and extraction. It is now specified that:*

*“All recorded trials were reviewed by the second author P.S. on image quality based on clarity and artifacts. Out of the three recorded trials, only the one with the best image quality would be selected for the subsequent image processing and data extraction.”*

*We humbly acknowledge that the variations in event sequence among the three recordings were not analysed in the present study. We have highlighted the issue under “Limitations and recommendations”:*

*“Considering the substantial amount of time and labour intensiveness required for data extraction, data were extracted from only one trial for each bolus type in each subject. It is recommended that data may be obtained from more trials to eliminate possible measurement and/or extraction errors, and to examine the extent of possible within-subject between-trial variability.”*

5) P8, L7 – In Fig. 1, the authors depicted the tongue base by green line, but the real “line of tongue surface was not clearly shown. Further, the authors are recommended to show all the pictures at time of TBOn, TBMax” and TBOff in one trial.

*Response:*

*Thank you for the suggestions. The tongue surface is now pointed with green arrows to clearly show the actual line of tongue surface in Fig 1.*

*In addition, Figs 2a, 2b, 3a, and 3b are added to show sequential frames of the respective events with onset, maximum, and offset highlighted by color frames, and overlays of the corresponding frames and landmark – tongue base is depicted with a line here to show comparisons between different states, and the hyoid bone is indicated by the center point of a bounding box.*

6) P20, Limitations – One of the biggest limitations is that the authors measured only the time of onset, peak (maximum) and offset of TB and HB. If was the movement just straight? How was the time duration of the tongue base staying at the maximum?

*Response:*

*Thank you for the very constructive comment. Since this study is primarily about the temporal sequences, and their adherence and variability, of the selected swallowing events; the duration aspect was not investigated. We have, nevertheless, highlighted the issue for readers at the end of the “Limitations and recommendations” section:*

*“Despite that duration of movements may be deduced from the differences in timing of events (e.g. the duration of maintaining maximum tongue base retraction may be deduced from the difference between TBMax and TBOff), analyses on movement duration and/or latency among events may be also be included in future studies.”*
